# Cloning and Functional Analysis of Lignin Biosynthesis Genes *Cf4CL* and *CfCCoAOMT* in *Cryptomeria fortunei*

**DOI:** 10.3390/genes10080619

**Published:** 2019-08-15

**Authors:** Zhenhao Guo, Hui Hua, Jin Xu, Jiaxing Mo, Hui Zhao, Junjie Yang

**Affiliations:** 1 Key Laboratory of Forest Genetics & Biotechnology of Ministry of Education, Nanjing Forestry University, Nanjing 210037, China; 2 Co-Innovation Center for Sustainable Forestry in Southern China, Nanjing forestry University, Nanjing 210037, China; 3 College of Forestry, Nanjing Forestry University, Nanjing 210037, China

**Keywords:** *Cryptomeria fortunei*, lignin synthetase, gene cloning, expression analysis, functional verification

## Abstract

*Cryptomeria fortunei*, also known as the Chinese cedar, is an important timber species in southern China. The primary component of its woody tissues is lignin, mainly present in secondary cell walls. Therefore, continuous lignin synthesis is crucial for wood formation. In this study, we aimed to discover key genes involved in lignin synthesis expressed in the vascular cambium of *C. fortunei*. Through transcriptome sequencing, we detected expression of two genes, *4CL* and *CCoAOMT*, known to be homologous to enzymes involved in the lignin synthesis pathway. We studied the function of these genes through bioinformatics analysis, cloning, vascular cambium expression analysis, and transgenic cross-species functional validation studies. Our results show that *Cf4CL* and *CfCCoAOMT* do indeed function in the pathway of lignin synthesis and likely perform this function in *C. fortunei*. They are prime candidates for future (gene-editing) studies aimed at optimizing *C. fortunei* wood production.

## 1. Introduction

*Cryptomeria fortunei* is a widely cultivated, strongly adaptable woody species that has a high economic value due to its rapid growth. Therefore we aim to improve our understanding of *C. fortunei* wood production by studying its lignin synthesis pathways. Wood is the secondary xylem of perennial woody plants that is formed as a result of the proliferation and differentiation of vascular cambium cells. It is mainly composed of cellulose, hemicellulose, and lignin, among which lignin is the main component of the secondary cell wall. It occupies approximately 25–35% [1] of the wood dry weight. Lignin can enhance the mechanical support of stems and provide resistance to bacterial infections.

Lignin is a phenylpropane complex composed of three kinds of alcohol monomers: coumarinol, coniferyl alcohol and sinapyl alcohol. Lignin can be classified into three separate categories, each composed of a different monomer: syringyl lignin (S-type lignin), guaiacyl lignin (G-type lignin), and para-hydroxy-phenyl lignin (H-type lignin). Previous studies have shown that in conifers lignin is mainly composed of G-type monomers [2], while primitive conifer species such as *Podocarpus macrophyllus* may contain S-type lignin monomers [3].

The biosynthesis of lignin is a complex process involving multiple enzymes. The synthetic route can be roughly divided into three steps. The first step is the synthesis of aromatic amino phenylalanine from photosynthetic assimilation products, called the shikimic acid pathway. In the second step, the phenylpropanoid pathway, different compounds are synthesized from phenylalanine, involving enzymes such as phenylalanine ammonia-lyase (*PAL*), cinnamate 4-hydroxylase (*C4H*), *p*-coumarate 3-hydroxylase (*C3H*), caffeic acid/5-hydroxyferulic acid *O*-methyltransferase (*COMT*), caffeoyl CoA *O*-methyltransferase (*CCoAOMT*), 4-(hydroxy) cinnamoyl CoA ligase (*4CL*), etc. The initial reaction of the phenylpropanoid pathway is catalyzed by *PAL*, which catalyzes the conversion of phenylalanine into cinnamic acid [4]. During the third step, a specific metabolic pathway involving the enzymes cinnamoyl CoA reductase (*CCR*) and cinnamyl alcohol dehydrogenase (C*AD*) leads to the synthesis of lignin monomers. Finally, various lignin monomers polymerized to construct lignin, catalyzed by peroxidase and laccase.

Kentaro et al. screened expressed sequence tags in *C. japonica* (Japanses cedar) cambium tissue using transcriptome sequencing [5]. *C. japonica* has a growing season from March to October, during which it shows lignification activity in its xylem tissues; activity sharply increases from March to June, then declines again until dormancy in October [5]. Kentaro et al. found the expression of most key enzymes involved in lignin and cell wall synthesis sharply increase from March to April and then slowly decline until dormancy. Interestingly, two lignification enzyme homologues, *PAL4* and *4CL3*, showed an opposite expression pattern, which increase during dormancy period [5]. This finding indicates that maybe not all such enzymes are involved in the main lignin synthesis needed for growth and some might act as a response to various stresses, such as infection, wounding, or drought.

In order to study the genetic basis of lignin synthesis in *C. fortunei* further, we selected *4CL* and *CCoAOMT*, homologues of two key lignin synthesis enzymes. The enzymes express strongly correlates to growth activity in both *C. japonica* and *C. fortunei*. The enzymes act in close succession of the lignin biosynthesis pathway. *4CL* catalyzes the conversion of compounds such as hydroxycinnamic acid and *p*-Coumaric acid into their corresponding CoA esters, allowing them to be the monomers during the biosynthesis of lignin [4]. Previous studies have shown that *4CL* is a member of a multi-gene family: *Pinus taeda* and *Petroselinum hill* possess two *4CL* gene family members [6], while *Nicotiana tabacum* and *Glycine max* contain more gene family members [7]. Not all *4CL* genes are involved in lignin synthesis: in *Populus trichocarpa*, the *Ptc4CL1* gene does contribute to lignin biosynthesis in xylem, whereas the *Ptc4CL2* gene participates in the biosynthesis of phenolic compounds, such as flavonoids, in epidermal cells [8,9,10,11].

*CCoAOMT*, another key enzyme involved in lignin synthesis, acts as a methyltransferase, catalyzing the conversion of Caffeoyl CoA into Feruloyl CoA [12]. Ye et al. first confirmed that *CCoAOMT* participates in lignin biosynthesis in the woody plant *Zinnia elegans* [13]. In pine, it plays a key role in G-type lignin synthesis [14]. Similar to *4CL*, *CCoAOMT* is part of a multi-gene family: For example, there are 1 to 2 *CCoAOMT* genes in *Medicago Sativa* Linn and *Populus spp*. [15,16] and 5 to 10 genes in *Zinnia elegans* Jacq. [13].

We demonstrated a functional analysis of *C. fortunei 4CL* and *CCoAOMT*, key enzymes involved in the lignin synthesis pathway. We performed a transcriptomic analysis of the vascular cambium [17] and found a large number of differentially expressed genes between growth stages and detected *4CL* and *CCoAOMT* among them. The overexpression of *Cf4CL* in *Arabidopsis* leaded to increased lignin deposition, strongly suggesting that it performs a similar function in *C. fortunei*, accelerating the formation of cedar wood and enhancing wood resistance. *CfCCoAOMT* is involved in the synthesis of G and S-type lignin; whether it is also involved in the biosynthesis of lignin and interacts with other key enzymes in the phenylpropanoid pathway remains to be further studied. Our results may be of significance towards the improvement of *C. fortunei* wood cultivation and properties.

## 2. Materials and Methods

### 2.1. Plant Material

For our experiments, we used *C. fortunei* growing in the arboretum of Nanjing Forestry University, aged around 50 years. Nanjing lies in the east of China, locates between 31°14′′ to 32°37′′ north latitude and 118°22′′ to 119°14′′ east longitude.The exact dates of sampling were 15 March, 15 May, 15 July, 15 September, and 15 November in 2017. Samples were marked with the date of collection, as we treated them as 5 successive growth stages of *C. fortunei* in one year. We collected vascular cambium zone composed of cambium, partial phloem and developing xylem by scratching the stem tissue with a carpet knife [18], collecting the tissue in a sterile enzyme-free 2 mL centrifuge tube and submerging it in liquid nitrogen, after which it was quickly stored at −80˚C. For genetic transformation and functional verification experiments, we used *Nicotiana tabacum*, a wild type tobacco strain with red flower.

### 2.2. Extraction of Total RNA and Synthesis of First Strand cDNA

Total *C. fortunei* vascular cambium RNA was extracted using an RNAprep^®^ Pure Plant Kit (Polysaccharides & Polyphenolics-rich, Tiangen Biotech Co., LTD, Beijing, China). We tested total RNA integrity and concentration using an EasyPure^®^ Quick Gel Extraction Kit (Beijing TransGen Biotech Co., Ltd, Beijing, China) and a Thermo Scientific NanoDrop 2000 (Thermo Fisher Scientific Co., Ltd, Waltham, MA, USA). We achieved cDNA through reverse transcription from total RNA using the PrimeScript™ RT Master Mix (Takara Biomedical Technology (Beijing) Co., Ltd, Beijing, China).

### 2.3. Molecular Cloning and Genetic Transformation of Tobacco

To determine the *Cf4CL* and *CfCCoAOMT* cDNA sequences, 3′ and 5′ race primers were designed based on the analysis of high-throughput sequencing data of the *C. fortunei* vascular cambium transcriptome (Table 1). The RACE reaction was carried out according to the manufacturer’s instruction using a SMARTer^®^ RACE 5′/3′Kit (Takara Biomedical Technology (Beijing) Co., Ltd, Beijing, China). Target segments were cloned into an Easy^®^-Blunt Cloning Vector (Beijing TransGen Biotech CO., Ltd, Beijing, China) and transformed into Trans1^®^-T1 Phage Resistant Chemically Competent Cells (Beijing TransGen Biotech CO., Ltd, Beijing, China). After selecting positive clones, they were sent to Nanjing GenScript Biotech Corp. (Nanjing, China) for sequencing. In order to verify the accuracy of the full-length sequence of the gene we designed primers outside the ORF and then performed PCR amplification verification. We designed primers to amplify the coding region of *4CL* and *CCoAOMT* using CE Design (Vazyme Biotech Co., Ltd, Nanjing, China) and tested the amplification products by 1% agarose gel electrophoresis. We constructed *4CL* and *CCoAOMT* overexpression vectors by linearizing and ligating the pBI121 vector with each insert using a ClonExpresss^®^ II One Step Cloning Kit C112 (Vazyme Biotech Co., Ltd, Nanjing, China). After sequence verification, the recombinant plasmid was transferred into agrobacterium EHA105 (Shanghai Weidi Biotechnology Co.,Ltd, Shanghai, China) using the freeze-thaw method. Transgenic plants were obtained using the tobacco leaf disc transformation referred to the method optimized by Curtis et al. [19], transgenic plants were identified by extracting transgenic tobacco DNA followed by PCR detection.

### 2.4. Bioinformatics Analysis

We used ProtParam [20] to analyze the physical and chemical properties of proteins encoded by *Cf4CL* and *CfCCoAOMT*. The number of amino acids, relative molecular weight, theoretical isoelectric point (PI value), protein formula, fat coefficient, hydrophilicity, and stability coefficient were taken as indices. After that, cDNA sequences of *Cf4CL* and *CfCCoAOMT* were blasted with the NCBI blastn web tool to find the most closely related known gene sequences from other plant species. We constructed multiple sequence alignments using DNAMAN 6 software (Lynnon Biosoft, San Ramon, CA, USA). The gene phylogenetic trees were constructed using MEGA 6.0 [21]. In addition, we used ProtParam online software to analyze the physical and chemical parameters of protein sequences, including relative molecular weight and theoretical isoelectric point. The NCBI CDS online tool was used to analyze gene-encoded protein domains and SOPMA software [22] used for secondary structure analysis and prediction. We used Swiss-Model Software [23] for analysis and construction of a protein tertiary structure model, and TMHMM v.2.0 software (http://www.cbs.dtu.dk/services/TMHMM/) to analyze the protein transmembrane region. Finally, we made use of ProtScale software [20] for protein hydrophobicity analysis and Signal IP4.0 [24] for the prediction of signal peptide sites.

### 2.5. Expression Analysis

Oligo7 [25] was used to design primers for qRT-PCR of *Cf4CL*, *CfCCoAOMT*, and the reference gene β-Actin (Table 1). The qRT-PCR was conducted using SYBR Premix Ex Taq™ II (Tli RnaseH Plus, TaKaRa Biomedical Technology (Beijing) Co., Ltd, Beijing, China), and ABI 7500 Step One Plus Real-time PCR platform [26]. Reactions were performed in biological triplicate, and six technical repetitions per sample and average Ct values were obtained for each gene at each growth stage. qPCR amplification data were processed with ΔΔCt. The relative quantitative data of the expression changes of three genes at five growth and development stages of *C. fortunei* vascular cambium formation were analyzed through SPSS v24 (IBM Co., Ltd, New York, NY, USA).

### 2.6. Transgenic Plant Growth and Phenotyping

Agrobacterium-infected tobacco leaf discs were incubated on MS selection medium (1.0 mg/L 6-BA, 0.1 mg/L NAA, 50 mg/L Kan, 200 mg/L TMT) and fostered for morphological observation at the stage of callus induction, adventitious bud growth and seedlings strengthened and root growth. Seedlings with strong roots were planted in the soil 5 days after adventitious roots appeared. The phenotype of 2-months-old mature tobacco plants, such as plant height, stem diameter, and leaf type were observed and compared. We chose 3 random seedlings of WT and both kinds of transgenic tobaccos for measuring. At the same stage, we observed the anatomical structure of stem segment cross sections with a Quanta 200 environmental scanning electron microscope (SEM) [27].

### 2.7. Determination of Lignin Content

The lignin content of mature tobacco was determined according to the acetyl bromide method improved by J. Rodrigues et al. [28], and data was collected using a GeneQuant pro ultraviolet (UV) spectrophotometer (Biochrom Ltd, Cambridge, UK).

## 3. Results

From previous work, we acquired transcriptome data from *C. fortunei* stem vascular cambium at different growth stages [17], and we identified genes expressed in this tissue. These genes can support the study involved with cambium development. Here, we chose *4CL* and *CCoAOMT* as strong candidate genes in lignin synthesis and performed bioinformatics and functional analysis to confirm their biological function.

### 3.1. *C. fortunei* Contains *Cf4CL* and *CfCCoAOMT* Genes

**Figure 1:**
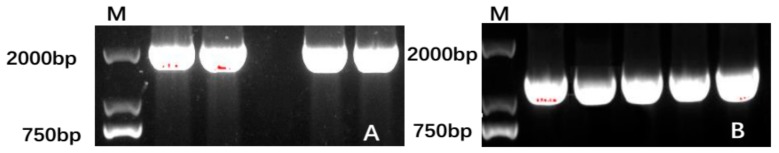
Amplification of the full length *Cf4CL* and *CfCCoAOMT* gene cDNA from stem vascular cambium total RNA. Primers used are listed in Table 1. Each lane represents a technical replicate. (**A**) *Cf4CL*; (**B**) *CfCCoAOMT*.

We obtained the 3′ and 5′ terminal sequence and intermediate fragment sequences of *Cf4CL* and *CfCCoAOMT* by sequencing. The *Cf4CL* intermediate fragment was 843 bp in length, and its full-length spliced cDNA was 2065 bp long, of which 1665 bp encoded 554 amino acids (Figure 1A). The *CfCCoAOMT* intermediate fragment was 685 bp in length, and its full-length spliced cDNA was 1219 bp long, of which 750 bp encoded 249 amino acids (Figure 1B).

### 3.2. *Cf4CL* and *CfCCoAOMT* Sequences Are Conserved

#### 3.2.1. Protein Sequence Multiple Alignment and Phylogenetic Tree Construction

**Figure 2:**
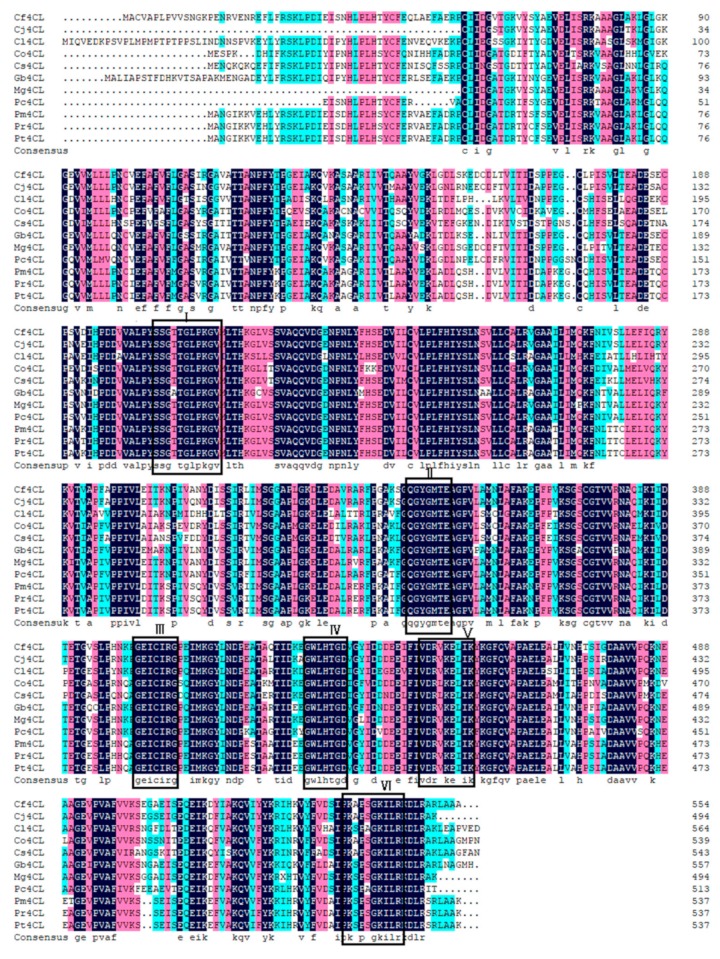
Multiple sequence alignment of the *4CL* protein using 10 plant species. The species represented by the abbreviations on the left side of the figure and their registration numbers in NCBI are as follows: *Cf4CL* (*C. fortunei*, MK236392), *Cj4CL* (*C. japonica*, AFJ73433.1), *Mg4CL* (*Metasequoia glyptostroboides*, AFJ73430.1), *Pc4CL* (*Pseudotaxus chienii*, AFJ73459.1), *Gb4CL* (*Ginkgo biloba*, AMN10098.1), *Pm4CL* (*Pinus massoniana*, ACO40513.1), *Pt4CL* (*P. taeda*, AAA92669.1), *Pr4CL* (*P. radiate*, ACF35279.1), *Cl4CL* (*Cunninghamia lanceolate*, AFX98059.1), *Co4CL* (*Cinnamomum osmophloeum*, AFG26323.1), *Cs4CL* (*Camellia sinensis*, ASU87411.1). The marked boxes are the six strongly conserved regions.

Through comparing the degree of sequence conservation of *4CL* and *CCoAOMT* sequences from multiple plant species, we could identify the nature of our cloned genes [29]. We found that the *4CL* amino acid sequence was highly conserved and could detect in total six (Box I-VI) highly conserved regions across the *4CL* sequence of all 10 chosen plant species (Figure 2) [30]. Box I (SSGTTGLPKGV) coded for an AMP binding domain required for the reaction catalyzed by *4CL*. *4CL* substrates, such as *p*-Coumaric acid, were converted into their corresponding CoA ester together with AMP. The reaction progressesed from the C- to N-terminus of the peptide chain, consumed ATP and used Mg^2+^ as cofactor [31]. Previous studies have shown that the C-residue found in Box III (GEICIRG), fully conserved across all species, was involved in the catalytic process, and its removal causes the *4CL* gene to be inactive [32]. Gly, Glu, and Cys were the most conserved amino acids. 6 highly conserved regions, SSGTTGLPKGV, QGYGMTE, GEICIRG, GWLHTGD, VDRLKELIK, and PKSPSGKILR, were discovered among all known *4CL* protein-coding sequences [30]. The amino acid sequence encoded by *4CL* of *C. fortunei* had the highest similarity (94%) to that of *C. japonica*. This high conservation confirmed that the gene cloned during the experiment to be a *4CL* gene and it was named as *Cf4CL*.

**Figure 3:**
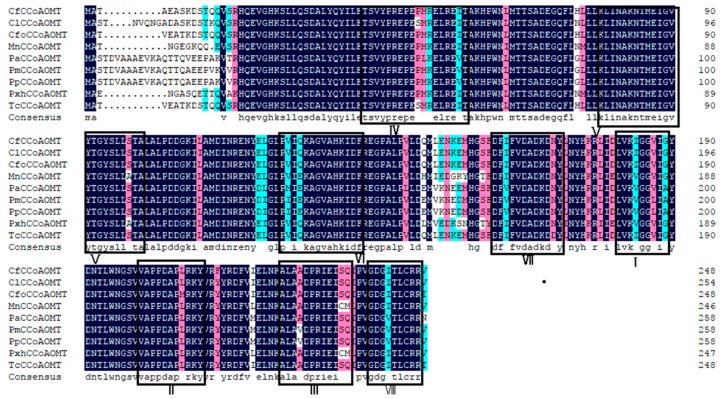
Multiple sequence alignment of the *CfCCoAOMT* protein using 9 plant species. The species represented by the abbreviations on the left side of the figure and their registration numbers in NCBI are as follows: *CfCCoAOMT* (*C. fortunei*, MK236394), *CfoCCoAOMT* (*Chamaecyparis formosensis*, ABB89956.1), *TcCCoAOMT* (*Taiwania cryptomerioides*, ABB87184.1), *ClCCoAOMT* (*Cunninghamialanceolata*, AFX98065.1), *CofCCoAOMT* (*Chamaecyparis obtusa var. formosana*, ABB87185.1), *PaCCoAOMT* (*Picea abies*, CAK18782.1), *PpCCoAOMT* (*P. pinaster*, AFL65039.1), *PmCCoAOMT* (*P. massoniana*, AHL67654.1), *AtCCoAOMT* (*Amborella trichopoda*, XP_006856484.1), *MnCCoAOMT* (*Morus notabilis*, XP_010107916.1), *PxhCCoAOMT* (*Petunia* x *hybrida*, ALP75648.1).

We then turned to the *CCoAOMT* protein sequence and found that it was equally highly conserved among 9 different plant species (Figure 3). Altogether, we found 8 conserved amino acid regions among all the known *CCoAOMT* protein sequences (Box I–VIII). Boxes I, II, and III represented plant methylase specific components, while the other 5 regions, Boxes IV–VIII, were unique to the *CCoAOMT* gene [30]. We found that all clones of the *C. fortunei CCoAOMT* gene contained these conserved areas, while mutations only emerged at a few sites. We found that the amino acid sequence encoded by the *C. fortunei CfCCoAOMT* gene was up to 98% similar to the *Chamaecyparis formosensis CCoAOMT* gene (Figure 3). The *Chamaecyparis formosensis CCoAOMT* gene has been annotated to catalyze the methylation of caffeoyl CoA in lignin biosynthesis based on its similarity to *CCoAOMT* genes discovered in *Oryza sativa* (ABB89956.1) [12]; it is possible that the *C. fortunei CCoAOMT* gene had a similar function. We therefore concluded that the cloned gene should be the homologue of *CCoAOMT* and named it *CfCCoAOMT*.

**Figure 4:**
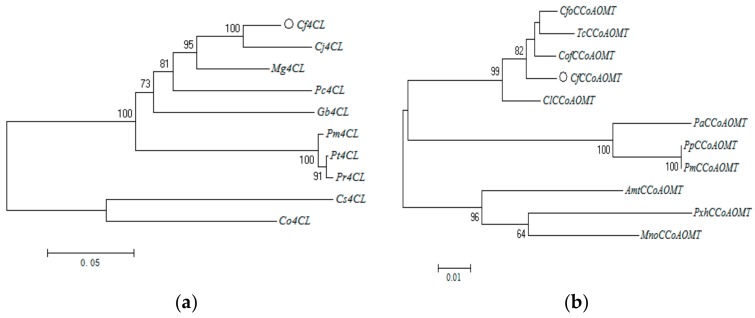
Phylogenetic trees of 4CL (**a**) and CCoAOMT (**b**) proteins using the most closely related protein sequences from 9 (**a**) and 10 (**b**) plant species. Displayed are tree diagrams of the interrelationships between the branches of each biological lineage, constructed according to their phylogeny to indicate the genetic relationship between species. Trees were generated using the unrooted Maximum Likelihood algorithm trees with MEGA 6.0 software. Bars represent genetic distance.

The phylogenetic tree of *4CL* was divided into 2 phylogenetic groups: one represented gymnosperms including the *4CL* gene of *C. fortunei*, *C. japonica*, *M. glyptostroboides*, *P. chienii*, *G. biloba*, *P. massoniana*, *P. taeda* and *P.radiate*, the other represented angiosperms including the *4CL* gene of *C. osmophloeum* and *C. sinensis* (Figure 4a). The *4CL* gene of *C. fortunei* had the highest similarity with that of *C.japonica*. The phylogenetic tree of *CCoAOMT* is divided into 3 phylogenetic groups: one included the *CCoAOMT* gene of *C. fortunei*, *Chamaecyparis formosensis*, *Chamaecyparis obtusa var. formosana*, *T. cryptomerioides*, and *Cunninghamia lanceolata*, the second included the *CCoAOMT* gene of *P. abies*, *P. pinaster* and *P. massoniana*, while the final group included the *CCoAOMT* gene of *A. trichopoda, M. notabilis and P.* x *hybrida* (Figure 4b). The *CCoAOMT* gene of *C. fortunei* was more similar to that of *Chamaecyparis formosensis*, *Chamaecyparis obtusa var. formosana* and *T. cryptomerioides*.

#### 3.2.2. *Cf4CL* and *CfCCoAOMT* Protein Structure and Function Prediction

We aimed to predict *Cf4CL* and *CfCCoAOMT* protein function. First, we analyzed the physicochemical properties of both *Cf4CL* and *CfCCoAOMT* using ProtParam online tools (Table 2).

The *Cf4CL* protein was composed of 554 amino acids, among which Ala took the largest part with a 9.9% occurrence, followed by Val (9.6%) and Leu (9.0%). The total average hydrophilicity is 0.124, and the instability coefficient was 36.15, indicating *Cf4CL* protein likely to be a stable, hydrophobic protein. The *CfCCoAOMT* protein was significantly smaller, been encoded by only 249 amino acids, with Leu taking the largest percentage of 11.6%, followed by Ala (7.6%) and Asp (7.2%).The same physicochemical parameters for *CfCCoAOMT* (Table 2) leaded us to conclude that it should also be a stable, yet hydrophilic, protein.

**Figure 5:**
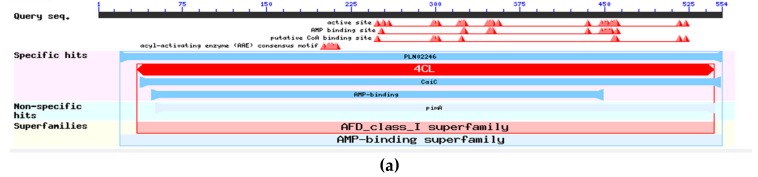

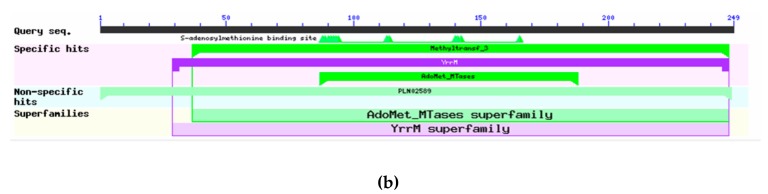
Protein domain analysis of Cf4CL (**a**) and CfCCoAOMT (**b**) proteins in *C. fortunei*. The figure was constructed with NCBI conserved domains. Specific hits are the top-ranking RPS-BLAST hits (compared to other hits in overlapping intervals) that meet or exceed a domain-specific E-value threshold (details and illustration). Non-specific hits meet or exceed the RPS-BLAST threshold for statistical significance. Superfamily is the domain cluster to which the specific and/or non-specific hits belong.

We then used NCBI conserved domains to analyze predicted protein domains in *Cf4CL* and *CfCCoAOMT*. Figure 5 indicates the individual *Cf4CL* and *CfCCoAOMT* protein domains. The *Cf4CL* protein contained an AMP binding site and was classified as a unique conserved “*4CL*” structural domain, belonging to the AFD-class-I family (Figure 5a). Within the *CfCCoAOMT* protein, we detected a methyltransferase domain (aa28-aa247), which belonged to the AdoMet-Mtases family (Figure 5b).

We used the online software package SOPMA to predict the secondary structures within the *Cf4CL* and *CfCCoAOMT* proteins (Table S1). We found that both proteins have a similar distribution of secondary structure types, with alpha helices and random coils being most frequent (Table S1). For the protein encoded by *Cf4CL*, the proportions occupied by alpha helix, random coil, extended strand, and beta turn were 30.87%, 35.56%, 24.37%, and 9.20%, respectively. For the protein encoded by *CfCCoAOMT*, the proportions occupied by alpha helix, random coil, extended strand, and beta turn were 37.35%, 32.53%, 20.88%, and 9.24%, in turn.

**Figure 6:**
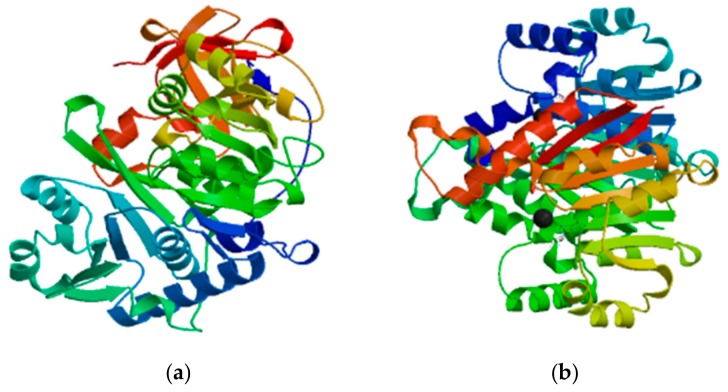
Tertiary structure model of the *C. fortunei Cf4CL* (**a**) and *CfCCoAOMT* (**b**) proteins. Color shows the order of peptide chain, starting as dark blue from the N-terminus, going to red at the C-terminus.

We then predicted the tertiary structure of the *Cf4CL* and *CfCCoAOMT* proteins using the online software Swiss-Model (Figure 6). It can be concluded from Table S1 that both proteins mainly consisted of alpha helices and random coils, corresponding well with the prediction of the secondary structures in Table S1. The results of protein hydrophobicity analysis through ProtScale showed that *Cf4CL* was a hydrophobic protein while *CfCCoAOMT* was hydrophilic (Figure S1). We predicted the transmembrane domain using TMHMM and found that *Cf4CL* and *CfCCoAOMT* both contained no transmembrane structure (Figure S2). In accordance with this result, using Signal IP, we found that both proteins did not contain any predicted signal peptide sequence (Figure S3).

### 3.3. Quantitative Real-time PCR Analysis of *Cf4CL* and *CfCCoAOMT*

**Figure 7:**
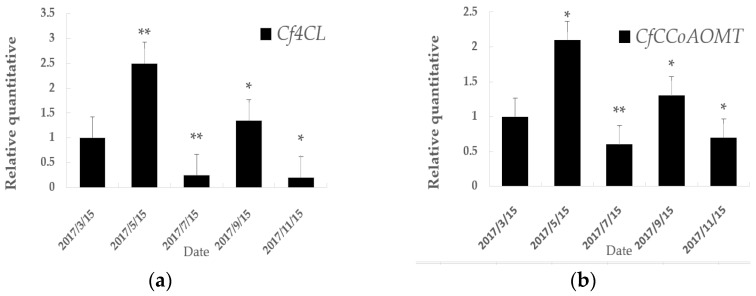
Expression of *Cf4CL* (**a**) and *CfCCoAOMT* (**b**) in *C. fortunei* vascular cambium during the different growth stages throughout the year. Plotted are means +/− standard deviation (*n* = 6 replications for each growing stage). *p* values were calculated with t-test, * *p* < 0.05, ** *p* < 0.01.

From our bioinformatics analysis we concluded that *Cf4CL* and *CfCCoAOMT* were the *C. fortunei* homologues of two enzymes involved in lignin synthesis, which were highly conserved across plant families. As lignin synthesis in trees was dependent on their seasonal growing activity [33], we aimed to analyze the relationship between the expression level of both genes and seasonal growing activity. Using qRT-PCR analysis of *Cf4CL* and *CfCCoAOMT* during different stages of growth and development, we found that expression of both genes could be detected during all five growth stages of the *C. fortunei* vascular cambium, yet with varying relative expression levels (Figure 7).

The expression level of *Cf4CL* increased significantly from March to May and peaked at mid-May, reaching its highest expression level (Figure 7a). This was followed by a rapid decline around mid-July, while a second, more gradual increase happened at mid-September. Finally, in mid-November, the expression dropped to its lowest level (Figure 7a). The expression level of *CfCCoAOMT* showed a very similar trend across the different growth stages, however the decline in expression in mid-July and mid-November was less sharp (Figure 7b).

### 3.4. Functional Analysis of *Cf4CL* and *CfCCoAOMT*

#### 3.4.1. *Cf4CL* and *CfCCoAOMT* May Increase Lignin Synthesis

**Figure 8:**
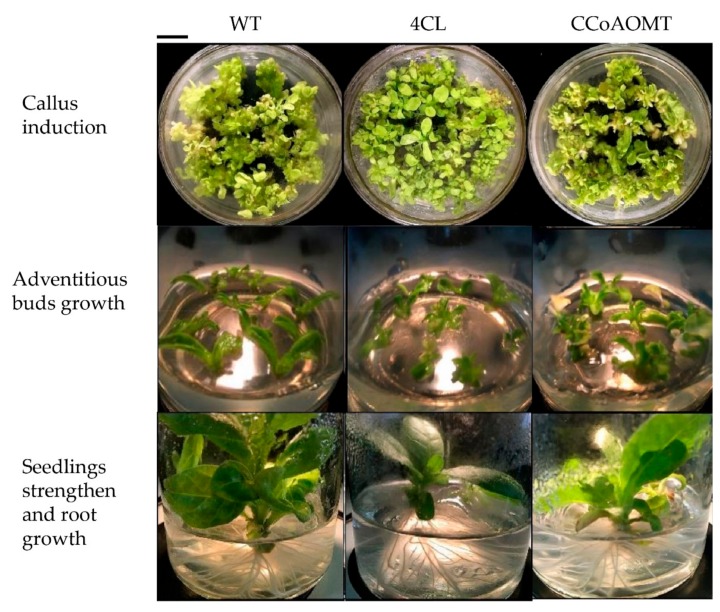
Overexpression of *Cf4CL* and *CfCCoAOMT* initially impedes tobacco growth. The first to third columns show representative images of wild type, *Cf4CL* transfected, and *CfCCoAOMT* transfected leaves, respectively. The first to third rows successively show the callus induction, adventitious bud growth, and seedling strengthening/root growth phases. A total of 3 seedlings were randomly chosen for measurements of each genotype. Bar equals 1 cm.

We next tested whether the *Cf4CL* and *CfCCoAOMT* genes can indeed affect lignin synthesis and plant growth in vivo, using a transgenic assay. We infected tobacco leaves using agrobacterium transformed with *35S::Cf4CL* or *35S::CfCCoAOMT* vector to induce high expression of either gene. Next, we placed transfected alongside control leaves on callus induction medium to study the resulting growth of regenerated plantlets. We found that wild type tobacco leaves grew faster than *Cf4CL* or *CfCCoAOMT* transfected leaves at every growth stage: callus induction, adventitious bud growth, seedling strengthening, and root growth (Figure 8, top to bottom). Moreover, the transgenic tobacco seedlings showed different degrees of yellowing, vitrification, and plant weakness.

It can be seen from Table S2 that the height difference of mature plants (2 months old) did not reach significance as the average height of wild type tobacco was 37.2 cm while *Cf4CL* and *CfCCoAOMT* transfected tobacco plants were 39.4 cm and 40.5 cm high, respectively. However, both transfected plants did develop a higher stem diameter, with the wild type of 4.92 mm, while *Cf4CL* and *CfCCoAOMT* transfected plants reached 5.68 mm (1.15× WT) and 6.35 mm (1.29× WT), respectively. Though growing slower than wild type at early growth stages, transgenic seedlings caught up and grew taller than the wild type, which might indicate a delayed function of *Cf4CL* and *CfCCoAOMT* in stem growth.

**Figure 9:**
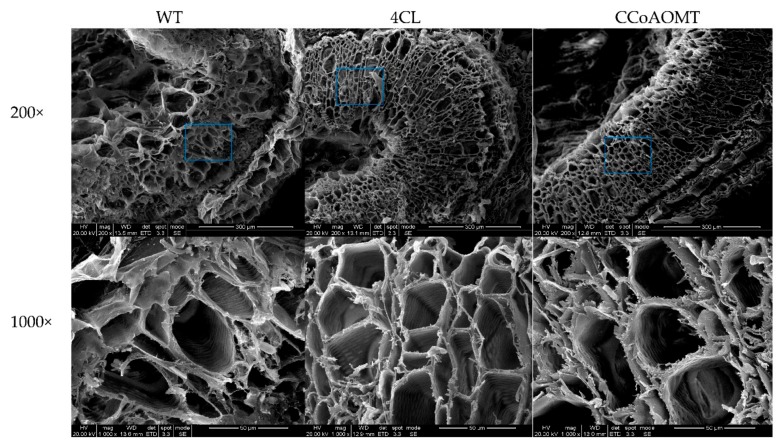
Representative SEM micrographs of tobacco stem cross sections, taken at 200× and 1000×. The first to third columns show wild type, *Cf4CL* and *CfCCoAOMT* transfected tobacco respectively. The pictures at 1000× show part of the pictures taken at 200×, indicated by a blue square.

Since we found that the stem diameter of *Cf4CL* and *CfCCoAOMT* transfected plants was increased, we wondered whether such plants might have an altered vascular cambium due to heightened lignin synthesis activity. We obtained cross sections of wild type and transfected tobacco stems as samples and soaked them in FAA for fixation. After dehydration with a series of alcohol solutions of increasing strength, samples were sliced and gilded. For further details, check the review of Brenda L. et al and article of Ma J. et al. [27,34]. We found that overexpression of either *Cf4CL* or *CfCCoAOMT* causes an increased number of xylem cells, a thickened xylem cell wall, and an increased number of cell layers in vascular tissues, compared to the wild type (Figure 9; Table S3). The average thickness of the xylem cell wall in three *Cf4CL* transgenic tobacco lines (*Cf4CL*-1, 2, 3) was 2.458 μm, 2.464 μm, and 2.488 μm, almost twice (~1.8×, *p* < 0.01) as thick as in the wild type (Table S3). We found similar values for three *CfCCoAOMT* transgenic lines (*CfCCoAOMT*-1, 2, 3), also being almost twice (~1.8×, *p* < 0.01) as thick as the wild type. These results showed that increased expression of *Cf4CL* and *CfCCoAOMT* might lead to a thickened xylem wall, most likely by increased lignin deposits.

#### 3.4.2. *Cf4CL* and *CfCCoAOMT* Expression Increases Lignin Content

To determine whether the thickened xylem walls we observed in our transgenic tobacco with higher levels of *Cf4CL* and *CfCCoAOMT* expression were caused by increased lignin content, we turned to directly measuring lignin levels of mature tobacco transferred into soil for 2 months using UV spectroscopy. Indeed, we found that in all transgenic lines analyzed, lignin content was significantly higher than in the wild type (Table 3). These results showed that overexpression of *Cf4CL* and *CfCCoAOMT* in tobacco leaded to an increase in lignin content, resulting in a thickened xylem cell wall. We therefore concluded that the *Cf4CL* and *CfCCoAOMT* genes we identified were indeed functional.

## 4. Conclusions and Discussion

In this study, we aimed to understand which genes might be involved in lignin synthesis in *C. fortunei* in order to understand lignin synthesis in this species and aim to improve its wood characteristics. We chose to study *4CL* and *CCoAOMT* as they are key enzymes in the phenylalanine pathway and have an important role in G-type or S-type lignin monomer biosynthesis. In most plant species, *4CL* genes exist as a gene family. It has been confirmed that the isoenzymes of *4CL* have different preferences for different substrates. Three *At4CL* genes were isolated from *A. thaliana*, and it was found that isoenzymes encoded by *At4CL1* and *At4CL2* are involved in the biosynthesis of lignin monomers [35]. Research has shown that the overexpression of *4CL* may accelerate the formation of wood and enhance the disease resistance and lodging resistance of wood [36,37]. *CCoAOMT* genes similarly exist as gene families in most plant species, showing high sequence conservation. They may work together in the biosynthesis of lignin with *COMT*, also a methylase, during which it may affect synthesis of both S-type lignin and G-type lignin [38].

We found that the *4CL* and *CCoAOMT* genes cloned from *C. fortunei* have high similarity with that of other species and confirmed with the inspiration of method performed by Wang et al. [39] *Cf4CL* and *CfCCoAOMT* may increase lignin synthesis and xylem wall thickness when overexpressed in tobacco, confirming their biological functionality. Even though tobacco overexpressing either *4CL* or *CCoAOMT* eventually grew taller than the wild type, initially their growth was delayed. Further study should show focus on whether this has to do with the presence of agrobacterium used for the transformation protocol. Further research needs to be carried out on whether the *CfCCoAOMT* gene contributes to lignin biosynthesis together with other key enzymes. We sampled *C. fortunei* vascular cambium at 5 successive growth stages to analyze the relative expression levels of *Cf4CL* and *CfCCoAOMT*. Our findings were consistent with previous studies on *Populus tomentosa 4CL* gene expression dynamics carried out by Zhao et al. [40] and on the *Populus tremuloides CCoAOMT* gene carried out by Meng et al. [41]. These and our study all showed a ‘double-peak’ pattern during the growing season, with the second expression peak being lower than the first. However, there appeard to be a difference between *C. fortunei* and the other two species in terms of the timing of the highest expression levels of *Cf4CL* and *CfCCoAOMT*. In *C. fortunei*, this occurred at mid-May and mid-September for both genes. In *P. tomentosa* the *4CL* gene peaked at late June and early August, while in *P. tremuloides* the *CCoAOMT* gene peaked around mid-June and late July. We speculate that this difference was due to variations between species and climatic conditions. Soile et al. analyzed the transcriptome data of *Picea abies* wood formation related genes and found that the seasonal expression of all lignin monomers peaked in summer and did not decrease until the end of August; the second peak was delayed to winter with the lowest temperature [18]. The expression levels of *4CL* and *CCoAOMT* also showed a ‘double-peak’ pattern, which is possible due to diverse climate zones and growth rhythms. Studies have confirmed that temperature change plays a key role in the formation of vascular cambium and the formation of late wood in conifer species [42]. Changes in photoperiod also affect the seasonal expression of genes involved in lignin synthesis in conifer species: Cronn R. et al. used RNA-seq to monitor transcriptional activity in *Pseudotsuga menziesii* (Douglas-fir) needles at daily and annual cycles and identified 12,042 transcripts that showed significant cyclic variation with changes in photoperiod, including transcripts involved in wood formation [28,43,44].

We found that the expression level of two *C. fortunei* genes involved in lignin synthesis decreased suddenly in mid-July below the average level. The average temperature in Nanjing at July is 27 °C to 34℃, peaks around the year, is harmful for most plants. We speculate that the reason for this decrease might be that *C. fortunei* experienced a higher temperature around July, longer illumination time and stronger transpiration, leading to inhibition of vascular formation by inhibiting cell differentiation division. Alternatively, certain internal regulatory factors may be affected during transcription or post-transcriptional regulation due to changes in the external environment, resulting in a change in RNA stability, slowing down the rate of lignin synthesis and resulting in the lower growth speed around mid-July [45]. Future experiments could include more sampling time points for a more accurate and detailed record of expression changes. At the same time, different tissues of *C. fortunei* can be sampled to study whether lignin synthesis might be differentially regulated in other tissues.

*C. fortunei* wood is of high value, having a rapid growth rate with a lower density of wood. We demonstrated here that *Cf4CL* and *CfCCoAOMT* are involved in lignin synthesis and subject to dynamic regulation of their expression, likely as the lignin build-up within *C. fortunei* changes throughout the season. Future studies are needed to confirm whether both genes indeed perform the same function in *C. fortunei* as well, for example, by constructing mutants or performing gene knockdown. Understanding how lignin synthesis operates in *C. fortunei* will help us to understand how its wood is formed, possibly allowing us to enhance its wood quality.

## Figures and Tables

**Table 1:** Primers used for partial cDNA cloning, RACE reaction, qRT-PCR.

Primer Name	Primer Sequence (5′-3′)	Application
Longer	CTAATACGACTCACTATAGGGCAAGCAGTGGTATCAACGCAGAGT	Universal primer
Shorter	CTAATACGACTCACTATAGGGC	
4CL-5′outer	GGCATTCGCTCTCATCCGCCTCAGTCAG	*4CL* RACE reaction
4CL-5′inner	GCTCCACCGGACATGATCAAGC	
4CL-3′outer	ATAGCCCACCTGAAGGTTGCCTCCCGATT	
4CL-3′inner	CTATTCCCTCAATTCGGTGCTC	
CCoAOMT-5′outer	GTATTGTCATAGCCGATCACTCCCCCAA	*CCoAOMT* RACE reaction
CCoAOMT-5′inner	CAATTTTGTGGGCAACACCTG	
CCoAOMT-3′outer	GCCCTGCCTGATGATGGAAAGATCCTAG	
CCoAOMT-3′inner	GATCGGCTATGACAATACTCTG	
4CL-ALL-F	GGGTAAAGATCAATTACTGCTTC	Amplification of the conserved region
4CL-ALL-R	CAAATTTATGTGTGCTGCGAGT	
CCoAOMT-ALL-F	GTTTCATTCCTCCAATCCAGT	
CCoAOMT-ALL-R	GGGCTGTTCTTAAATCACTCC	
RT-4CL-F	CTCCTTTGTGCGCTCCGAGT	RT-PCR
RT-4CL-R	GCTCCACCGGACATGATCAAGC	
RT-CCoAOMT-F	GCTCATCAATGCCAAGAACACCA	
RT-CCoAOMT-R	TCAATTTTGTGGGCAACACCT	
CE-4CL-F	ATGGCTTGTGTCGCACCTCT	Enzyme cutting
CE-4CL-R	TTAGGCTGCTGCAAGTCTGGC	
CE-CCoAOMT-F	ATGGCAACTGCAGAGGCTTC	
CE-CCoAOMT-R	AATAACTCTTCTGCAGAGAGTGATGC	

**Table 2:** Analysis of protein physical and chemical properties.

	Amino Acids/aa	Molecular Weight/kD	PI Value	Molecular Formula	Fat Coefficient	Hydrophilicity	Unstable Coefficient
*Cf4CL*	554	59.86	5.41	C_2700_H_4309_N_699_O_793_S_19_	103.84	0.124	36.15
*CfCCoAOMT*	249	28.098	5.52	C_1261_H_1994_N_338_O_372_S_8_	99.88	−0.228	39.40

**Table 3:** Determination of the content of lignin in tobacco.

Plant	WT	*4CL*			*CCoAOMT*		
		1	2	3	1	2	3
Average lignin content %	4.86	8.31	10.51	7.14	9.13	7.54	8.34
SD	0.983	1.457	2.819	0.404	2.318	0.507	1.365
Sig		<0.05	<0.01	<0.05	<0.01	<0.05	<0.01

For every tobacco genotype 3 plants were measured (*n* = 3), 3 technical repetitions were done per plant sample. SD, standard deviation from selected lines. Sig, the level of significance, data was compared through *t*-test.

## Supplementary Materials

The following are available online at https://www.mdpi.com/2073-4425/10/8/619/s1. Table S1: Analysis of secondary structure of encoded protein; Table S2: Comparison of plant height and stem diameter after Tobacco Maturity; Table S3: Transverse section cell wall thickness of tobacco stem segments; Figure S1: Hydrophobicity curves of the *Cf4CL* and *CfCCoAOMT* gene encoding protein of *C. fortunei*; Figure S2: Transmembrane pattern map of *Cf4CLC* and *fCCoAOMT* gene encoding protein of *C. fortunei*; Figure S3: Pattern diagram of protein signal peptide encoded by *C. fortunei Cf4CL* and *CfCCoAOMT* gene.
