# Supplementary material for: Cloning and Functional Analysis of Lignin Biosynthesis Genes Cf4CL and CfCCoAOMT in Cryptomeria fortunei
Source: Genes (Basel). 2019 Aug 15;10(8):619. doi: 10.3390/genes10080619 (PMC6723087; doi:10.3390/genes10080619)
Supplement: Supplementary file 1 [file genes-10-00619-s001.pdf]

genes

Supplementary Materials

Table S1. Analysis of secondary structure of encoded protein.

Gene	Alpha helix	Random coil	Extended strand	Beta turn
Cf4CL	30.87	35.56	24.37	9.21
CfCCoAOMT	37.35	32.53	20.88	9.24

Table S2. Comparison of plant height and stem diameter after Tobacco Maturity.

Plant	WT	4CL	CCoAOMT
Plant height/cm	37.17<br>5	39.36<br>7	40.5
SD	6.214	3.659	5.188
Sig		0.556	0.442
Stem diameter/mm	4.92	5.68	6.35
SD	0.717	0.465	0.662
Sig		<0.05	<0.01

For every tobacco genotype 3 plants were measured (n=3). SD, standard deviation from selected lines. Sig, the level of significance, data were compared through T-TEST.

Table S3. Transverse section cell wall thickness of tobacco stem segments.

Plant	WT	*4CL*			*CCoAOMT*		
		**1**	**2**	**3**	**1**	**2**	**3**
	**(*n* = 30)**	**(*n* = 32)**	**(*n* = 30)**	**(*n* = 30)**	**(*n* = 32)**	**(*n* = 32)**	**(*n* = 31)**
Cell wall thickness/μm	1.365	2.458	2.464	2.488	2.565	2.501	2.472
SD	0.225	0.295	0.260	0.298	0.287	0.264	0.244
Sig		<0.01	<0.01	<0.01	<0.01	<0.01	<0.01

For every tobacco genotype 3 plants were measured (*n* = 3). SD, standard deviation from selected lines. Sig, the level of significance, data were compared through T-TEST

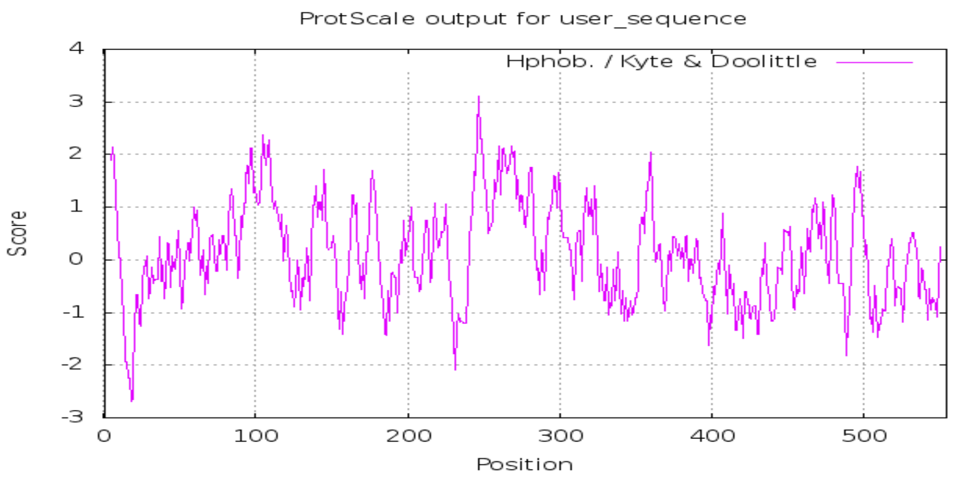

(a)

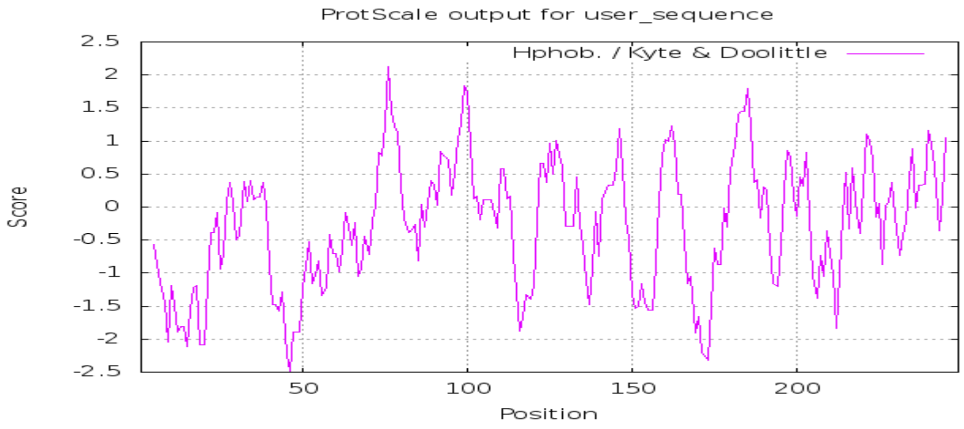

(**b**)

Figure S1. Hydrophobicity curves of the *Cf4CL* and *CfCCoAOMT* gene encoding protein of *C. fortunei*. (**a**) *Cf4CL* (**b**) *CfCCoAOMT*.

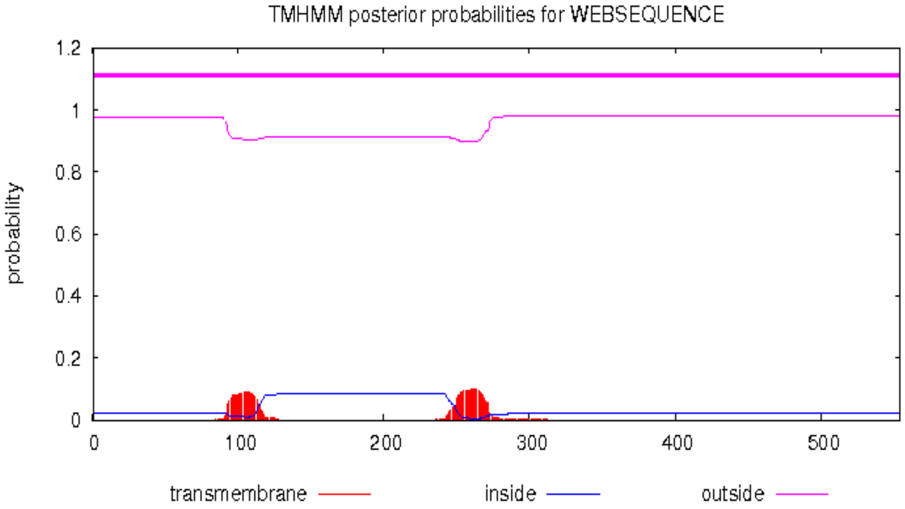

(**a**)

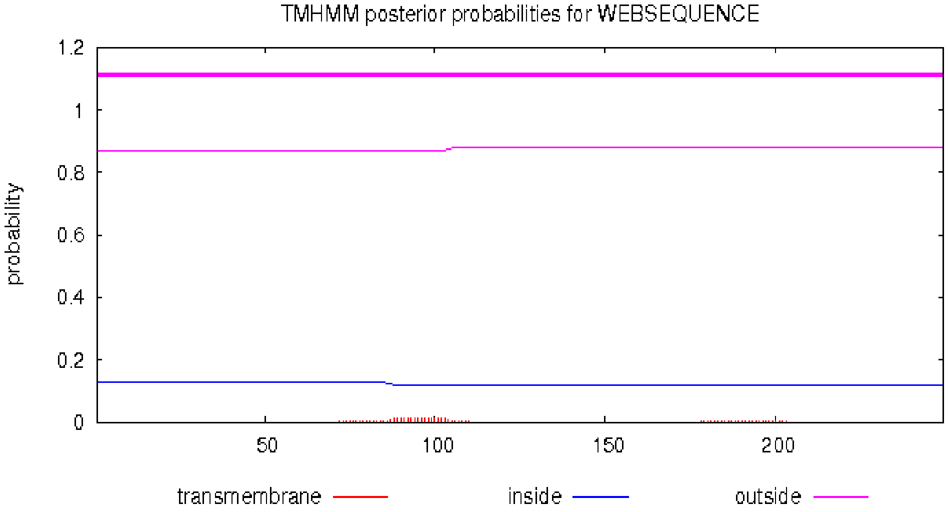

(**b**)

Figure 2. Transmembrane pattern map of *Cf4CLC* and *fCCoAOMT* gene encoding protein of *C.fortunei*. (**a**) *Cf4CL*, (**b**) *CfCCoAOMT*.

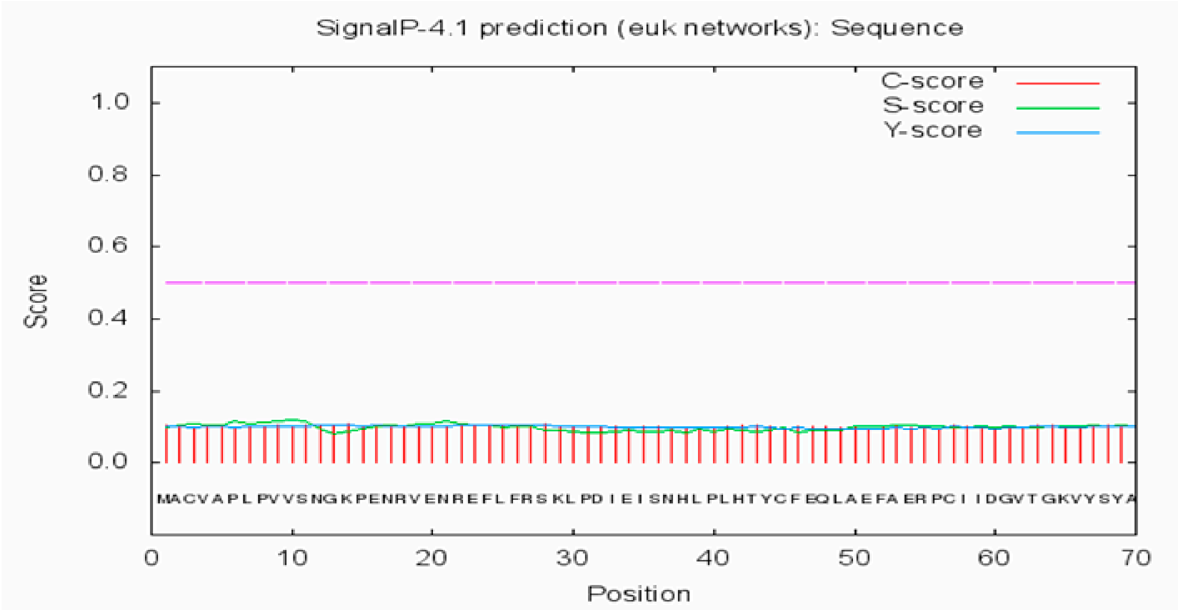

(**a**)

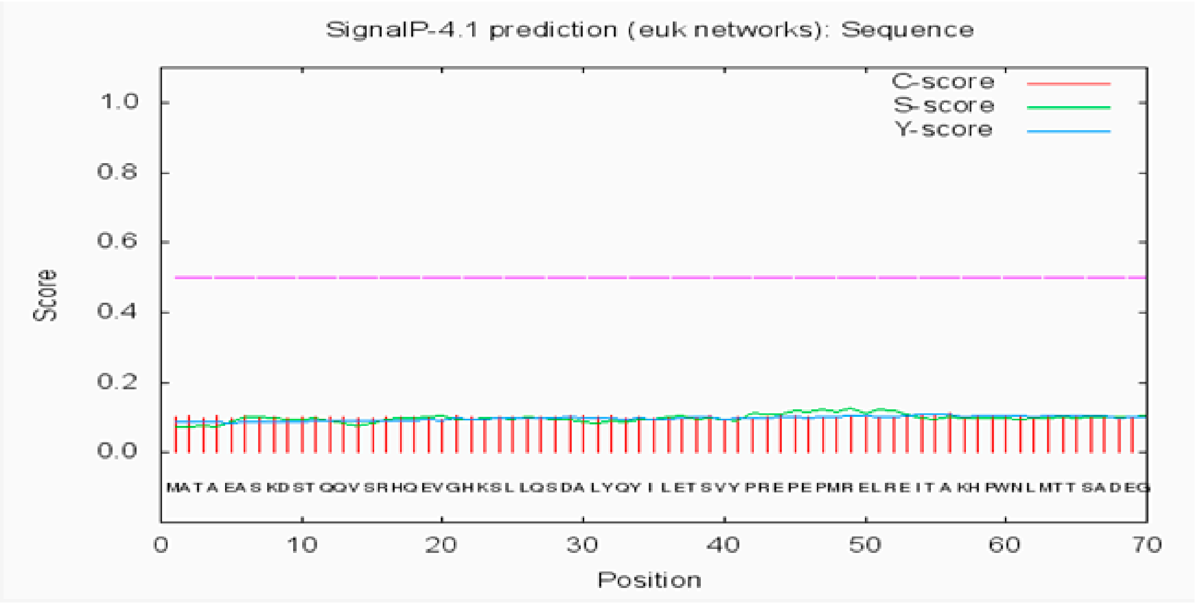

(**b**)

Figure S3. Pattern diagram of protein signal peptide encoded by *C. fortunei Cf4CL* and *CfCCoAOMT* gene. (**a**) *Cf4CL,* (**b**) *CfCCoAOMT.*
